# Association between depression and antibiotic use: analysis of population-based National Health Insurance claims data

**DOI:** 10.1186/s12888-021-03550-2

**Published:** 2021-10-28

**Authors:** Jong-Wook Lee, Hankil Lee, Hye-Young Kang

**Affiliations:** 1 College of Pharmacy, Yonsei Institute of Pharmaceutical Sciences, Yonsei University, 85 Songdogwahak-ro, Yeonsu-gu, Incheon, 21983 South Korea; 2 Graduate Program of Industrial Pharmaceutical Science, Yonsei University, Incheon, South Korea; 3 College of Pharmacy, Ajou University, Suwon, South Korea

**Keywords:** Antibiotics, Infectious disease, Major depressive disorder

## Abstract

**Background:**

Frequent exposure to antibiotic treatments may increase the risk of antibiotic resistance, which may threaten the effectiveness of future antibiotic treatments. Thus, it is important to identify the preventable risks in terms of antibiotic use. This study assessed the association between major depressive disorder (MDD) and antibiotic use by comparing the likelihood and extent of antibiotic use between patients with and without MDD.

**Methods:**

This retrospective cross-sectional study utilized the National Patients Sample data from the 2017 Health Insurance Review and Assessment Service. We analyzed 16,950 patients with MDD, defined as those with at least two claims records stating a primary diagnosis of MDD (International Classification of Diseases, 10th revision codes F32–33) and 67,800 patients without MDD (1:4 propensity-score matched control group). Antibiotic use was compared between the patients with and without MDD based on three variables: the presence of antibiotic prescriptions, total prescription days of antibiotics per year, and total medication costs of antibiotics per year.

**Results:**

The adjusted odds ratio obtained by multivariate regression analysis for the presence of prescription of antibiotics was 1.31 (95% confidence interval [CI]: 1.25–1.36). In the negative binomial model, the number of prescription days was 1.25 times (95% CI: 1.23–1.28) higher in patients with MDD than in those without MDD. Generalized linear model analysis showed a 1.39-fold (95% CI: 1.36–1.43) higher cost of antibiotic prescription in patients with MDD than in those without MDD.

**Conclusions:**

Our results suggest a potential association between MDD and the prescription of antibiotics, implying that patients with MDD are relatively vulnerable to infections. It is important to prevent as well as closely monitor the occurrence of infections when managing patients with MDD.

**Supplementary Information:**

The online version contains supplementary material available at 10.1186/s12888-021-03550-2.

## Background

Depression is a common and serious medical illness characterized by a mood disorder with the following symptoms: a feeling of sadness, loss of interest or pleasure in daily matters, difficulty in falling asleep, feeling of worthlessness, and thoughts of death or suicide [1]. A steady increase in the number of patients with depression imposes a substantial burden on the society. Globally, the number of patients with depression increased by 17.8% from 183,434,000 in 2005 to 216,047,000 in 2015 [2]. Depression was predicted to be among the top 15 causes of disability-adjusted life years in 2019 [3]. Therefore, knowledge regarding the complications and comorbid conditions of patients with depression may assist in the appropriate management of these patients and in reducing the burden on healthcare.

Depression is not merely an emotional disorder, but it is also associated with a high risk of infectious diseases (ID) [4]. Previous studies have reported that major depressive disorder (MDD) can increase the inflammatory response due to elevated production of pro-inflammatory cytokines, such as interleukin-1 (IL-1), IL-6, and interferon-gamma [5, 6]. This chronic inflammatory response may alter the immune system, leading to increased susceptibility to infectious agents [7, 8]. Additionally, patients with depression may be less aware of their health conditions, such as infections, or may seek treatment less proactively [9]. Moreover, even if they start treatment for infection, they may recover more slowly from infections and require more aggressive antibiotic treatment as compared to the general population. Due to these physiological characteristics, patients with depression may be vulnerable to ID, thus becoming very likely to receive antibiotic treatments.

Antibiotic prescription is one of the most common medical interventions for the treatment of ID and can be used as a good indicator of the type and severity of the ID. Frequent exposure to antibiotic treatments may increase the potential for antibiotic resistance, which may result in poor effectiveness of antibiotic treatments in the future, and thus delayed cure. Hence, it is important to identify the preventable risks of antibiotic use and to develop proactive strategies to minimize these risks effectively.

This study aimed to assess the association between depression and antibiotic use by comparing the likelihood and extent of antibiotic use between patients with and without MDD using population-based real-world data. Furthermore, we compared the types of ID frequently treated with antibiotics and the types of antibiotics frequently prescribed for the two groups to identify the target ID requiring close monitoring in patients with MDD.

## Methods

### Study patients and data source

We used the data from the 2017 Health Insurance Review and Assessment Service–National Patient Sample (2017 HIRA-NPS, serial number: HIRA-NPS-2017-0044), which are nationally representative cross-sectional data comprising a random sample of 3% of the total population of South Korea (approximately 1,473,084 patients). The two-tiered National Health Security (NHS) system in South Korea comprises the National Health Insurance (NHI), in which the individuals contribute, and the subsidized public assistant Medical Aid (MA). The HIRA-NPS data include claim records for both NHI and MA beneficiaries and provide information regarding the diagnosis, diagnostic and medical procedures, and prescription drugs provided during medical treatment. Moreover, the data also include selective patient characteristics, such as age and sex, selective provider characteristics, such as the medical specialty of the clinicians, and the type and geographic location of the healthcare institutions. The study protocol was approved by the Institutional Review Board of Yonsei University, Seoul, South Korea (IRB No. 7001988–202,102-HR-1095-01E), which waived the requirement of informed consent from the study participants.

Patients with MDD were defined as those with at least two claim records with a primary diagnosis of MDD (International Classification of Diseases, 10th Revision [ICD-10] codes F32.x [major depressive disorder, single episode] or F33.x [major depressive disorder, recurrent]). Since the target population of the MDD group in this study was adult patients with MDD without severe comorbid conditions, we first excluded those aged below 20 years and then excluded those with claim records for cancer, end-stage renal disease, or transplants. The non-MDD group included patients with no claim records that indicated a diagnosis of MDD. From both groups, we excluded patients with records of antibiotic use during surgery, as this would involve prophylaxis against infections. After applying the inclusion and exclusion criteria, we identified 16,950 patients with MDD and 1,136,823 patients without MDD from among 1,180,910 adults aged 20 years or older in the 2017 HIRA-NPS. We then performed 1:4 propensity score (PS) matching [10]. We used the patients’ demographic characteristics, such as sex, age, and the type of NHS program enrolled, as the variables in greedy matching, which is a method that produces balanced matched samples [11]. In estimating the risk differences, a caliper width of 0.108 was used, which is equal to 0.2 multiplied by the standard deviation of the logit of the PS, to minimize the mean squared error [12]. The standardized difference of the mean was calculated as follows:

$$ d=\frac{\left({p}_1-{p}_2\right)}{\sqrt{\frac{p_1\left(1-{p}_2\right)+{p}_2\Big(1-{p}_{1\Big)}}{2}}} $$

where, p_1_ and p_2_ represent the prevalence of dichotomous variables in the MDD and non-MDD groups, respectively [13].

**Fig. 1:**
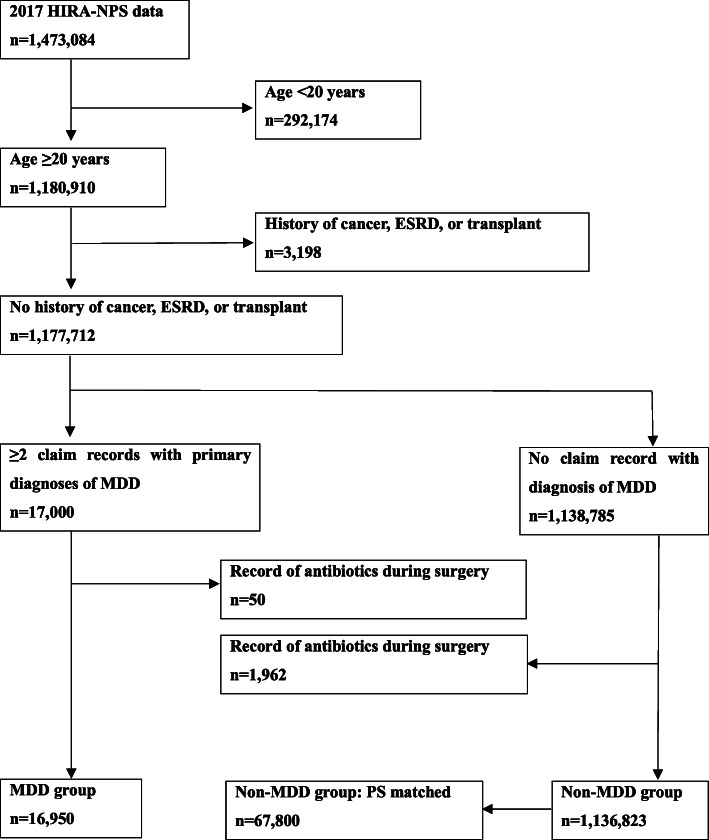
Flow chart of patient inclusion in the study. *n*, number of patients; *ESRD*, end-stage renal disease; *HIRA-NPS*, Health Insurance Review and Assessment–National Patient Sample; *MDD*, major depressive disorder; *PS*, propensity score

Figure 1 presents the flowchart of patient inclusion in this study.

### Antibiotic use

Antibiotic use was operationalized using three variables: presence of antibiotic prescriptions, total prescription days of antibiotics per year, and total medication costs of antibiotics per year. If a patient had at least one claim record of prescribed antibiotics in 2017, the variable for prescription of antibiotics was denoted as “1.” The prescription of antibiotics was included regardless of the patient’s treatment type, i.e., both the inpatient and outpatient use of antibiotics were included. For patients with a claim record of prescribed antibiotics, the total number of prescription days and total medication costs of antibiotics during 2017 were computed to examine the magnitude of antibiotic use among those receiving antibiotic therapy. Our analysis considered those antibiotics that had been approved and were covered by the NHI in Korea. Additional File 1 presents the specific antibiotics included in our analysis.

### Data analysis

Odds ratios (ORs) were calculated for comparing the presence of antibiotic prescriptions between the MDD group and PS-matched non-MDD group. Wilcoxon’s rank-sum tests were performed to compare the median prescription days and antibiotic costs between the two groups with patients receiving antibiotics. We developed three regression analysis models for each variable of antibiotic use to assess whether there were significant differences in the antibiotic use between the MDD and non-MDD groups. To compare the outcomes across different age groups, each analysis was performed for patients aged 20–49, 50–64, 65–74, and ≥ 75 years.

A logistic regression model was used to determine whether the patients were prescribed antibiotics at least once in 2017. Since the total prescription days of antibiotics per year were an over-dispersed count variable (mean ± standard deviation: 23.4 ± 43.6 for the MDD group and 15.7 ± 33.8 for the non-MDD group), the Poisson regression model or the negative binomial model were more appropriate than the standard least squares regression model [14, 15]. In the Lagrange multiplier test [16] of the over-dispersion factor alpha, the *p*-value was < 0.0001, which rejected the Poisson model in favor of the negative binomial model. Therefore, we designed a negative binomial regression model to examine the association between depression and the total prescription days of antibiotics among those who had been prescribed antibiotics once or more. Because the total medication costs of antibiotics per year were not normally distributed but had a skewed distribution with a long right tail, we performed a modified Park test to determine the distribution type for the variable. The model depended on the λ, which was the exponential of the raw-scale prediction in the relational expression with raw-scale variance. Since the λ estimate was 2.35, which approaches 2, we designed a generalized linear model with a gamma distribution [17].

The major independent variable was the presence of MDD. Selected patient characteristics, such as age, sex, and the type of NHS (NHI or MA), which were available in the claims data and were considered to be associated with antibiotic use, were adjusted by the PS matching method. The regression model also included selected comorbid conditions known to increase the risk of ID [18, 19]. If a patient had at least one claim record with a primary or secondary diagnosis of comorbid diseases, we assumed that the patient had the disease. Among respiratory diseases, we excluded those caused by bacterial infections, such as acute upper respiratory infections (J00–J06), influenza and pneumonia (J09–J18), and acute lower respiratory tract infections (J20–J22). Since the presence of these diseases is an underlying cause of antibiotic use, it was not appropriate to treat it as a confounder in the association between depression and antibiotic use. We used Statistical Analysis System version 9.4 (SAS Institute, Inc., Cary, NC, USA) to analyze the data.

## Results

### Characteristics of patients with and without depression

This study included 16,950 patients with MDD and 1,136,823 without MDD before the PS matching. The male-to-female ratio of the MDD group was approximately 1:2 (5579 males and 11,371 females). The proportion of patients aged ≥65 years in the MDD group (32.81%) was approximately twice that of the non-MDD group (16.86%). The MDD group patients had a lower economic status than did the non-MDD group patients: 13.66% patients of the MDD group were beneficiaries of the subsidized public assistant MA program compared to only 3% of the non-MDD group. The prevalence rates of all investigated comorbid diseases were significantly higher in patients with MDD than in patients without MDD. In particular, the prevalence rates of cerebrovascular disease (2.5-fold higher) and congestive heart failure (2.4-fold higher) were substantially higher in patients with MDD than in those without MDD (Table 1).

**Table 1:** Comparison of characteristics between patients with and without major depressive disorder

	Before PS^a^ matching			After PS^a^ matching			
	No. patients (%)		*p*-value^b^	No. patients (%)		*p*-value^b^	Standardized difference of mean (%)
	With MDD	Without MDD		With MDD	Without MDD		
Total no. of patients	16,950	1,136,823	–	16,950	67,800		
Sex							
Male	5579 (32.91)	553,585 (48.70)	< 0.0001	5579 (32.91)	22,316 (32.91)	1.0	0.000
Female	11,371 (67.09)	583,238 (51.30)		11,371 (67.09)	45,484 (67.09)		0.000
Age groups							
20–49 years	6207 (36.62)	622,844 (54.79)	< 0.0001	6207 (36.62)	24,828 (36.62)	1.0	0.000
50–64 years	5182 (30.57)	322,265 (28.35)		5182 (30.57)	20,728 (30.57)		0.000
65–74 years	2969 (17.52)	112,019 (9.85)		2969 (17.52)	11,876 (17.52)		0.000
≥75 years	2592 (15.29)	79,695 (7.01)		2592 (15.29)	10,368 (15.29)		0.000
NHS program enrolled							
NHI	14,635 (86.34)	1,102,761 (97.00)	<.0001	14,635 (86.34)	58,540 (86.34)	1.0	0.000
MA	2315 (13.66)	34,062 (3.00)		2315 (13.66)	9260 (13.66)		0.000
Comorbidities							
Diabetes	2949 (17.40)	113,602 (9.99)	< 0.0001	2949 (17.40)	9390 (13.85)	< 0.0001	9.762
Peripheral vascular disease	1012 (5.97)	30,454 (2.68)	< 0.0001	1012 (5.97)	2745 (4.05)	< 0.0001	8.801
Cerebrovascular disease	1368 (8.07)	37,440 (3.29)	< 0.0001	1368 (8.07)	3330 (4.91)	< 0.0001	12.797
Myocardial infarction	117 (0.69)	5236 (0.46)	< 0.0001	117 (0.69)	401 (0.59)	0.1398	1.238
Congestive heart failure	301 (1.78)	8445 (0.74)	< 0.0001	301 (1.78)	744 (1.10)	< 0.0001	5.699
Non-alcoholic fatty liver disease	490 (2.89)	19,458 (1.71)	< 0.0001	490 (2.89)	1247 (1.84)	< 0.0001	6.916
Obesity	40 (0.24)	990 (0.09)	< 0.0001	40 (0.24)	50 (0.07)	< 0.0001	4.125
Respiratory disease	6265 (36.96)	290,372 (25.54)	< 0.0001	6265 (36.96)	18,969 (27.98)	< 0.0001	19.098
Rheumatoid arthritis	361 (2.13)	11,804 (1.04)	< 0.0001	361 (2.13)	880 (1.30)	< 0.0001	6.406

^a^Patients without MDD were matched with patients with MDD by propensity score based on demographic characteristics, i.e., age, sex, and the type of NHS program enrolled

^b^Chi-square tests across the MDD and non-MDD groups

*MA* medical aid, *MDD* major depressive disorder, *NHI* National Health Insurance, *NHS* National Health Security, *PS* propensity score

After 1:4 PS matching for the demographic characteristics of patients, the number of patients in the non-MDD group decreased to 67,800. The standardized mean difference of the demographic characteristics was equal to 0, indicating that the covariates were well-balanced between the two groups [13].

### Antibiotic use

Antibiotics were prescribed 1.44 times more often for patients with MDD (OR: 1.44, 95% confidence interval [CI]: 1.38–1.50) than for those without MDD (PS-matched control group). The median number of the antibiotic prescription days per year among the patients with MDD was significantly higher than that of the patients without MDD (13 vs. 9 days; *p* < 0.0001). The median antibiotic prescription cost was also significantly higher in the MDD group than in the non-MDD group (13.50 vs. 9.51 US dollars; *p* < 0.0001) (Table 2).

**Table 2:** Comparison of antibiotic use between patients with and without major depressive disorder

	Presence of claim records of prescribed antibiotics		Total annual prescription days of antibiotics per patient with claim records of prescribed antibiotics		Total annual medication costs of antibiotics per patient with claim records of prescribed antibiotics (US dollars^a^)	
	Per 10,000 patients	Crude OR (95% CI)	Median (Q1, Q3)	*p*-value	Median (Q1, Q3)	*p*-value
PS-matched patients without MDD^b^ (*n* = 67,800)	7208	- (reference)	9 (4, 18)	–	9.51 (4.25, 20.72)	–
Patients with MDD (*n* = 16,950)	7881	1.44 (1.38, 1.50)	13 (6, 27)	<.0001^c^	13.50 (5.53, 31.14)	< 0.0001^c^

^a^1 US dollar approximately equals 1200 Korean won

^b^Patients without MDD were matched with patients with MDD by propensity score based on demographic characteristics, i.e., age, sex, and the type of National Health Security program enrolled

^c^Results of Wilcoxon rank sum tests compared with patients without MDD

*CI* confidence interval, *MDD* major depressive disorder, *OR* odds ratio, *PS* propensity score, *Q1* 25th percentile, *Q3* 75th percentile

The top 10 principal diagnoses of claim records of patients who were prescribed antibiotics were similar between the MDD and non-MDD groups, although some diagnoses differed between them (Table 3). For example, the primary diagnoses of “other diseases of the urinary system” (ICD-10 codes: N30–N39) and “chronic lower respiratory tract diseases” (J40–J47) were more common in the patients with MDD than in those without MDD (11.37% vs. 7.54 and 9.47% vs. 7.85%, respectively). The number of prescription days and the antibiotics costs for 6 of the 10 principal diagnoses were more in the MDD group than in the non-MDD group (Table 3). However, the types of antibiotics prescribed were similar between the two groups (Table 4).

**Table 3:** Comparison of frequent primary diagnoses in claims records with prescribed antibiotics

Rank	Patients with MDD (*n* = 16,950)				PS-matched patients without MDD^a^ (*n* = 67,800)			
	Description (ICD-10 codes)	% of patients	Median Rx days (Q1, Q3)	Median drug costs (Q1, Q3), (US dollar)	Description (ICD-10 codes)	% of patients	Median Rx days (Q1, Q3)	Median drug costs (Q1, Q3), (US dollar)
1	Other acute lower respiratory infections (J20-J22)	26.19	6 (3, 10)^***^	5.48 (3.28, 10.60)^***^	Other acute lower respiratory infections (J20-J22)	24.67	5 (3, 8)	4.78 (3.03, 8.83)
2	Diseases of oral cavity and salivary glands (K00-K14)	24.93	5 (3, 8)	2.65 (1.18, 5.48)^*^	Diseases of oral cavity and salivary glands (K00-K14)	23.85	5 (3, 8)	2.65 (0.95, 5.30)
3	Acute upper respiratory infections (J00-J06)	22.25	5 (3, 9)^***^	4.78 (2.87, 9.08)^***^	Acute upper respiratory infections (J00-J06)	20.26	4 (3, 8)	4.36 (2.68, 8.24)
4	Other diseases of the upper respiratory tract (J30-J39)	15.53	6 (3, 11)^***^	5.72 (3.18, 11.53)^**^	Other diseases of the upper respiratory tract (J30-J39)	13.54	5 (3, 9)	5.04 (3.09, 10.1)
5	Other diseases of the urinary system (N30-N39)	11.37	7 (4, 14)^***^	6.43 (3.29, 13.54)^***^	Chronic lower respiratory diseases (J40-J47)	7.85	5 (3, 9)	5.48(3.28, 10.18)
6	Chronic lower respiratory diseases (J40-J47)	9.47	6 (3, 12)^***^	6.57 (3.48, 14.2)^***^	Disorders of eyelid, lacrimal system and orbit (H00-H05)	7.79	3 (1, 5)	5.25 (2.80, 8.41)
7	Infections of the skin and subcutaneous tissue (L00-L08)	8.83	5 (3, 9)	4.26 (2.29, 8.24)	Disorders of conjunctiva (H10-H11)	7.70	1 (1, 3)	3.05 (2.46, 5.64)
8	Disorders of eyelid, lacrimal system and orbit (H00-H05)	8.59	3 (1, 5)*	4.68 (2.80, 8.22)*	Other diseases of the urinary system (N30-N39)	7.54	7 (4, 12)	5.73 (3.18, 11.28)
9	Inflammatory diseases of female pelvic organs (N70-N77)	8.48	6 (3, 11)^***^	3.04 (0.94, 6.82)^***^	Infections of the skin and subcutaneous tissue (L00-L08)	7.44	5 (3, 8)	4.26 (2.63, 7.81)
10	Disorders of conjunctiva (H10-H11)	8.33	1 (1, 3)	3.37 (2.59, 5.98)	Inflammatory diseases of female pelvic organs (N70-N77)	6.07	5 (2, 9)	2.29 (0.48, 5.67)

For each diagnosis, a Wilcoxon rank sum test was performed to compare the MDD patients with those without MDD

1 US dollar equals approximately 1200 Korean won

*p < 0.05; **p < 0.001; ***p < 0.0001

^a^Patients without MDD were matched with patients with MDD by propensity score based on demographic characteristics, i.e., age, sex, and the type of National Health Security program enrolled

*ICD* International Classification of Diseases, *MDD* major depressive disorder, *PS* propensity score, *Q1* 25th percentile, *Q3* 75th percentile, *Rx* prescription

**Table 4:** Comparison of the type of prescribed antibiotics between patients with and without major depressive disorder

Rank	Patients with MDD (*n* = 16,950)		PS-matched patients without MDD^a^ (*n* = 67,800)	
	Antibiotic type	Proportion (%)	Antibiotic type	Proportion (%)
1	Cephalosporins	32.65	Cephalosporins	31.98
2	Penicillins	21.88	Penicillins	24.17
3	Quinolones	19.11	Quinolones	18.14
4	Aminoglycosides	15.37	Aminoglycosides	14.58
5	Macrolides	5.20	Macrolides	5.59
6	Tetracyclines	2.44	Tetracyclines	2.24
7	Sulfonamides	1.05	Sulfonamides	1.21
8	Antituberculosis	0.84	Antituberculosis	1.11
9	Carbapenems	0.59	Carbapenems	0.38
10	Glycopeptides	0.47	Glycopeptides	0.32
11	Others	0.35	Others	0.28
12	Oxazolidinones	0.03	Oxazolidinones	0.02
13	Monobactams	0.02	Monobactams	0.01
Total		100		100

^a^Patients without MDD were matched with patients with MDD by propensity score based on demographic characteristics, i.e., age, sex, and the type of National Health Security program enrolled

*MDD* major depressive disorder, *PS* propensity score

### Analysis of the association between MDD and antibiotic prescriptions

Multivariate logistic regression analysis revealed a 1.31-fold higher risk of antibiotic treatment in the patients with MDD (95% CI: 1.25–1.36) than in those without MDD (PS-matched control group). In the negative binomial regression analysis, the number of antibiotic prescription days was 1.25 times (95% CI: 1.23–1.28) higher in patients with MDD than in those without MDD. In the generalized linear model analysis, the cost of antibiotic prescriptions was 1.39 times (95% CI: 1.36–1.43) higher in the MDD group than in the non-MDD group (Table 5).

**Table 5:** Results of regression analysis of the association between major depressive disorder and antibiotic use

	Use of antibiotics, OR (95% CI)	Days of antibiotic prescription, Factor change^a^ (95% CI)	Drug costs of antibiotics, Factor change^a^ (95% CI)
Depression			
Without MDD^b^	- (reference)	- (reference)	- (reference)
With MDD	1.31 (1.25–1.36)	1.25 (1.23–1.28)	1.39 (1.36–1.43)
Comorbidities			
Diabetes	1.14 (1.09–1.20)	1.25 (1.23–1.28)	1.56 (1.52–1.61)
Peripheral vascular disease	1.40 (1.29–1.53)	1.10 (1.06–1.14)	0.98 (0.93–1.02)
Cerebrovascular disease	0.98 (0.92–1.05)	1.23 (1.19–1.28)	2.27 (2.18–2.37)
Myocardial infarction	0.97 (0.79–1.19)	1.27 (1.14–1.40)	2.17 (1.92–2.47)
Congestive heart failure	1.04 (0.90–1.22)	1.34 (1.25–1.43)	2.86 (2.62–3.12)
Non-alcoholic fatty liver disease	1.41 (1.25–1.60)	1.21 (1.15–1.28)	1.41 (1.32–1.50)
Obesity	1.52 (0.86–2.68)	1.16 (0.93–1.46)	0.87 (0.66–1.16)
Respiratory disease	3.49 (3.35–3.64)	1.63 (1.60–1.66)	1.66 (1.62–1.69)
Rheumatoid arthritis	1.50 (1.29–1.74)	2.55 (2.40–2.71)	1.29 (1.19–1.39)
Dispersion parameter		0.965 (0.955–0.976)	
*p*-value for LM test^c^		< 0.0001	

Each of the regression models was adjusted by demographic characteristics, such as age, sex, and the type of National Health Security program enrolled, using a propensity score matching method

^a^Exp (coefficient estimate) = Factor change in expected prescription days and drug costs for a unit increase in a covariate

^b^Patients without MDD were matched with patients with MDD by propensity score based on demographic characteristics, i.e., age, sex, and the type of National Health Security program enrolled

^c^Lagrange multiplier (LM) test of the over-dispersion factor alpha

*CI* confidence interval, LM test Lagrange multiplier test, *MDD* major depressive disorder, *OR* odds ratio

### Subgroup analysis according to age group

**Fig. 2:**
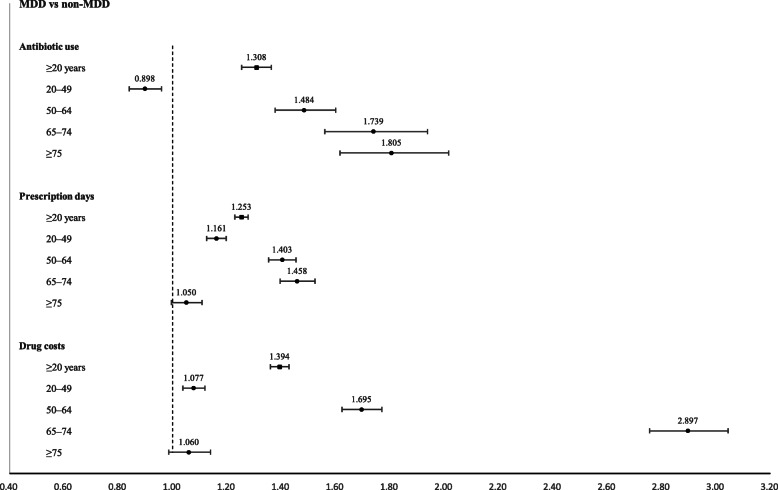
Regression analysis of the association between major depressive disorder and antibiotic use in different age groups. Each of the regression models were adjusted by demographic characteristics, i.e., age, sex, and the type of National Health Security program, using a propensity score matching method, and by comorbidities. Numerical values on the horizontal lines are the adjusted odds ratios, and the width of the horizontal lines are the associated 95% confidence intervals. *MDD*, major depressive disorder

The subgroup analysis according to the four age groups (20–49, 50–64, 65–74, and ≥ 75 years) showed that as the age increased, the positive association between MDD and the risk of antibiotic treatment also increased. Additionally, the positive association between MDD and the number of antibiotic prescription days and the antibiotic prescription costs increased with age; however, it was not significantly associated with the older age group (≥75 years) (Fig. 2).

## Discussion

This population-based epidemiological study investigated the association between MDD and antibiotic use. We compared the characteristics of antibiotic use between the MDD and non-MDD groups to assess depression as a risk factor for increased utilization of healthcare resources. Our hypothesis that MDD is associated with a high risk of antibiotic use was confirmed using three variables: presence of antibiotic prescriptions, antibiotic prescription days, and antibiotic medication costs (Table 5).

Although there was no marked difference in the types of frequent ID between the two groups, the antibiotic prescription days and medication costs for the same ID were higher in patients with MDD than in those without MDD. This implies that patients with MDD may experience more severe infections with similar ID and may require a longer treatment time as compared to those without. The most frequently administered types of antibiotics were almost identical between the MDD and non-MDD groups, which could be because the most frequent types of ID were also similar between the two groups.

The subgroup analysis for different age groups showed that the positive association between MDD and the risk of antibiotic use increased with age (Fig. 2). For young and middle-aged adults (i.e., 20–49 years), the likelihood of receiving antibiotic treatment was not higher in the MDD group when compared with the non-MDD group (OR: 0.898). However, among the patients in their 50s, a significantly higher risk of antibiotic use was observed in the MDD group than in the non-MDD group (OR: 1.484), which increased to an OR of 1.805 for those aged ≥75 years. This finding suggests the need for close monitoring to prevent ID in older patients with MDD. The positive associations between MDD and antibiotic prescription days and drug costs strengthened with age in those aged below 74 years. However, in the oldest age group (≥75 years), MDD was neither significantly associated with the number of antibiotic prescription days nor with the antibiotic costs. This implies that in older patients treated with antibiotics, MDD might not be a contributing factor influencing prolonged antibiotic treatment or high antibiotic costs.

Depression increases the production of several inflammatory cytokines, including IL-1, IL-6, and interferon-gamma [5, 6]. IL-6 activates the hypothalamic-pituitary-adrenal axis activity and increases the plasma concentrations of adrenocorticotropic hormone and cortisol, which induce multiple adverse immunological changes that make patients with depression susceptible to infection [20]. Depression also induces direct adverse effects on the immune system, including downregulation of the cellular and humoral responses [21]. Moreover, people with depression may be less aware of their health conditions, such as infections, or may be less proactive about treatment [9]. They are also likely to be susceptible to several diseases through unhealthy habits such as lack of sleep, alcoholism, drug abuse, poor nutrition, and reduced exercise [22]. Since patients with depression are more susceptible to ID and lack a healthy lifestyle as compared to those without depression, their use of antibiotics and the total prescription days will tend to be higher. Therefore, patients with depression will incur higher antibiotic costs as compared to the general population. However, in the literature, there is conflicting evidence regarding the causal relationship between MDD and antibiotic exposure or ID. Some studies have shown an association between antibiotic use and subsequent depression. For example, a nested case-control study reported that antibiotic treatment was associated with a high risk of depression (adjusted OR: 1.23 [95% CI: 1.18–1.29] for penicillins and 1.25 [95% CI: 1.15–1.35] for quinolones) [23]. Children and adolescents with enterovirus infection with central nervous system involvement had a 1.62-fold higher risk of depression than that of the control group (adjusted hazard ratio: 1.62, 95% CI: 1.02–2.58) [24]. Other studies have shown an inverse causal association. For example, a prospective cohort study reported that maternal depressive symptoms during pregnancy predicted significantly high rates of acute otitis media in infants [25]. Alvarodo-Esquivel et al. reported approximately twice as many cases of *Toxoplasma gondii* infection among patients with depression than in those without depression (OR: 2.14; 95% CI: 1.00–4.59) [26]. Our study provided evidence regarding the association between MDD and antibiotic use. However, because this was only a 1-year long cross-sectional study, we were unable to identify the temporal associations between MDD onset and antibiotic treatment.

This study has several limitations. First, because only patients with MDD with insurance claim records were included in the study, our analysis does not reflect the healthcare utilization behaviors of patients with undiagnosed or untreated MDD. Notably, people tend to search for diagnoses and treatments for psychiatric conditions, including MDD, less actively than for other conditions because they are concerned about social taboo and the potential disadvantages of patients with psychiatric conditions [27, 28]. Thus, the extent of under-diagnosis may be greater for MDD than for other diseases. Second, our study may have included inappropriate prescriptions of antibiotics. Fleming-Dutra et al. reported that only 69.8% of antibiotic prescriptions were appropriate, with the remaining prescriptions considered unnecessary [29]. Another study reported the appropriate use of antibiotics in 59.7% of patient prescriptions [30]. Since we could not distinguish between the inappropriate and appropriate prescriptions using the claims data, our study may have included inappropriate prescriptions.

## Conclusion

In summary, our analysis demonstrated a significant association between MDD and the risk of receiving antibiotic treatment, suggesting the susceptibility of patients with MDD to ID. Higher utilization of healthcare resources in terms of antibiotic prescription days and drug costs was observed in patients with MDD than in those without MDD for the same type of ID. Finally, the risk of being treated with antibiotics increased with age in the MDD group as compared to the non-MDD group. Thus, efforts should be made to prevent new episodes of depression in the older population.

## Supplementary Information

**Additional file 1 **List of antibiotics included in the analysis**.** This table describes the World Health Organization-anatomical therapeutic chemical classification system (WHO-ATC) codes and antibiotic types included in the analysis of this study

## Data Availability

The authors used data from the HIRA-NPS for this study and do not have permission to share these data. Researchers can request data access from the Health Insurance Review and Assessment Service (http://opendata.hira.or.kr).

## Abbreviations

CI: confidence interval
HIRA-NPS: Health Insurance Review and Assessment Service-National Patient Sample data
ICD-10: International Classification of Diseases, 10th revision
ID: infectious diseases
IL: interleukin
MA: medical aid
MDD: major depressive disorder
NHI: National Health Insurance
OR: odds ratio
PS: propensity score
