# Supplementary material for: Association between depression and antibiotic use: analysis of population-based National Health Insurance claims data
Source: BMC Psychiatry. 2021 Oct 28;21:536. doi: 10.1186/s12888-021-03550-2 (PMC8554858; doi:10.1186/s12888-021-03550-2)
Supplement: Supplementary file 1 — Additional file 1 List of antibiotics included in the analysis. This table describes the World Health Organization-anatomical therapeutic chemical classification system (WHO-ATC) codes and antibiotic types included in the analysis of this study [file 12888_2021_3550_MOESM1_ESM.docx]

**Additional File 1. List of antibiotics included in the analysis**

| Antibiotic types | Specific drugs (WHO-ATC code) |
| --- | --- |
| Penicillins | amoxicillin (J01CA04), amoxicillin/clavulanate (J01CR02), ampicillin (J01CA01), ampicillin/sulbactam ([J01CR01](https://www.druginfo.co.kr/p/product-search/atc/?atcCode=J01CR01)), nafcillin (J01CF06), penicillin G potassium crystal (J01CE01), benzathine penicillin G (J01CE08), piperacillin (J01CA12), piperacillin/tazobactam (J01CR05), ticarcillin/clavulanate (J01CR03) |
| Cephalosporins | cefaclor (J01DC04), cefadroxil (J01DB05), cefamandole (J01DC03), cefazolin (J01DB04), cefdinir (J01DD15), cefditoren (J01DD16), cefepime (J01DE01), cefixime (J01DD08), cefoperazone/sulbactam (J01DD62), cefotaxime (J01DD01), cefpodoxime (J01DD13), cefprozil (J01DC10), ceftazidime (J01DD02), ceftibuten (J01DD14), ceftizoxime (J01DD07), ceftriaxone (J01DD04), cefuroxime (J01DC02), cephalexin (J01DB01) |
| Monobactam | aztreonam (J01DF01) |
| Macrolides | azithromycin (J01FA10), clarithromycin (J01FA09), erythromycin (J01FA01, S01AA17) |
| Aminoglycosides | amikacin (J01GB06), gentamicin (J01GB03, D06AX07, S01AA11), kanamycin (J01GB04), neomycin (S01AA03, D06AX04), netilmicin (J01GB07), streptomycin (J01GA01), tobramycin (J01GB01, S01AA12) |
| Quinolones | ciprofloxacin (J01MA02), gatifloxacin (S01AE06), gemifloxacin (J01MA15), levofloxacin (J01MA12), lomefloxacin (J01MA07), moxifloxacin (J01MA14, S01AE07), ofloxacin (J01MA01, S01AE01, S02AA16) |
| Tetracyclines | doxycycline (J01AA02, A01AB22), minocycline (J01AA08), tetracycline (J01AA07), tigecycline (J01AA12) |
| Carbapenem | doripenem (J01DH04), ertapenem (J01DH03), imipenem/cilastatin (J01DH51), meropenem (J01DH02) |
| Sulfonamides | sulfadiazine (J01EC02, D06BA01), sulfamethoxazole/trimethoprim (J01EE01), sulfasalazine (A07EC01) |
| Oxazolidinones | linezolid (J01XX08), tedizolid (J01XX11) |
| Antituberculosis | cycloserine (J04AB01), ethambutol (J04AK02), isoniazid (J04AC01), pyrazinamide (J04AK01), rifampicin (J04AB02), isoniazid/rifampicin (J04AM02), isoniazid/rifampicin/ethambutol/pyrazinamide (J04AM06) |
| Glycopeptides | teicoplanin (J01XA02), vancomycin (J01XA01, A07AA09) |
| Others | chloramphenicol (S01AA01), clindamycin (J01FF01, G01AA10), fosfomycin (J01XX01) |

*WHO-ATC* World Health Organization-anatomical therapeutic chemical classification system
